# *Carica papaya* L. sex chromosome review and physical mapping of the *serk 2*, *svp-like* and *mdar 4* sequences

**DOI:** 10.1038/s41598-024-65880-x

**Published:** 2024-06-27

**Authors:** Adeilson Frias Dornela, Fernanda Aparecida Ferrari Soares, Jéssica Coutinho Silva, Mariana Cansian Sattler, Wellington Ronildo Clarindo

**Affiliations:** 1 Pós-Graduação em Genética e Melhoramento, Centro de Ciências Agrárias e Engenharias, Universidade Federal do Espírito Santo, Alegre, ES 29.500-000 Brazil; 2 Laboratório de Citogenética e Citometria, Departamento de Biologia Geral, Centro de Ciências Biológicas e da Saúde, Universidade Federal de Viçosa, Viçosa, MG 36.570-900 Brazil

**Keywords:** Papaya, Cytogenomics, Cytomolecular marker, Fluorescent in situ hybridization, Plant sex differentiation, Cytogenetics, Plant genetics

## Abstract

Physical mapping evidences the chromosome organization and structure. Despite the data about plant cytogenomics, physical mapping has been conducted from single-copy and/or low-copy genes for few species. *Carica papaya* cytogenomics has been accomplished from BAC-FISH and repeatome sequences. We aimed to map the *serk 2*, *svp-like* and *mdar 4* sequences in *C. papaya*. The sequences were amplified and the amplicons sequenced, showing similarity in relation to *serk 2*, *svp-like* and *mdar 4* genes. *Carica papaya* diploidy was confirmed and the mitotic chromosomes characterized. The chromosome 1 exhibited the secondary constriction pericentromeric to the centromere of the long arm. So, we concluded that it is the sex chromosomes. *serk 2* was mapped in the long arm interstitial portion of the sex chromosomes, and the interphase nuclei showed two fluorescence signals. Considering these results and the sequencing data from the *C. papaya* sex chromosomes, *svp-like* and *mdar 4* genes were mapped in the interstitial region of the sex chromosome long arm. Both sequences showed only one fluorescence signal in the interphase nuclei. The procedure adopted here can be reproduced for other single-copy and/or low-copy genes, allowing the construction of cytogenetic maps. In addition, we revisited the cytogenomics data about *C. papaya* sex chromosomes, presenting a revised point of view about the structure and evolution to these chromosomes.

## Introduction

*Carica papaya* L. (Caricaceae) originated in Mesoamerica. This species is an important commercial fruit cultivated in tropical and subtropical regions of the world^1^. Seminal propagation is the main system used for *C. papaya* cultivation, resulting in individuals of three biological sexes typically following a Mendelian inheritance^2^. *Carica papaya* possesses a relatively small nuclear genome size showing ~ 1C = 0.32 pg, equivalent to ~ 318 Mbp^3^. This species is diploid with karyotype exhibiting 2n = 2x = 18 chromosomes, which have been classified as metacentric and submetacentric^3,4^.

Sex chromosome evolution and sexual differentiation mechanisms have been investigated from different plant species, and *C. papaya* stands out as an important model species^5^. Sex differentiation in *C. papaya* was initially based on one gene with at least three alleles (*M*_1_, *M*_2_ and *m*). Considering this proposal, male, hermaphrodite and female plants differentiated from the genotypes *M*_1_* m*, *M*_2_* m* and *m m*, respectively^6,7^. The genotypes *M*_1_* M*_1_, *M*_2_* M*_2_ and *M*_2_* M*_1_ are lethal, promoting the zygotic embryo death^6,7^. Storey revised his hypothesis, suggesting that the sex is not differentiated by the expression of one gene with three alleles, but by linked genes located in a region of the sex chromosomes where recombination is suppressed^8^. Corroborating to this Storey’s hypothesis, further evidence showed that the recombination is severely suppressed in the region close to the sexual differentiation locus^9^, and at least seven genes were identified in the sexual specific portion^10^. Furthermore, structural chromosomal rearrangements (inversions, deletions and translocations), which may be the outcomes of the recombination suppression, were detected in the sex differentiation region^10^.

Sex differentiation region in *C. papaya* genome was genetically mapped to linkage group 1, which is related to the chromosome 1^11,12^. *C. papaya* biological sex is expressed by a XY chromosome sex differentiation system, from which females are homogametic (XX), and males (XY) and hermaphrodites (XYh) are heterogametic. Y and Yh chromosomes have a specific sexual differentiation region that shows few genetic differences^13^. Male-specific region (MSY) and hermaphrodite-specific region (HSY) are about 9.8 Mbp, while X-specific region (XX) has 6.0 Mbp^14^. MSY and HSY chromosome portions have a larger genome size in relation to the corresponding region on the X chromosome, mainly due to the DNA sequence duplications and insertion of mobile elements (retrotransposons) in this region^13^. HSY possesses more repetitive sequences than X. In addition, 121 pseudoautosomal genes occur between HSY and X chromosomes, 56 specific HSY genes, and 74 specific X genes^14^.

In association to cytogenomics, genomic sequencing data makes possible the physical mapping in mitotic and meiotic chromosomes of different DNA sequences, including single-copy and/or low-copy genes. Fluorescent in situ hybridization (FISH) is a cytogenomics technique that maps DNA sequences on chromosomes. FISH contributes to the construction of physical maps, and, consequently, the integration of these maps with genetic maps^15,16^. FISH in *C. papaya* 'Solo' and 'Maradol' mitotic chromosomes from 18S and 5S rDNA sequences evidenced the 18S rDNA in the pericentromeric portion of one chromosome pair, while the 5S rDNA was mapped in the pericentromeric portion of three chromosome pairs^17^. In contrast, pachytene chromosomes of *C. papaya* 'SunUp' showed 5S rDNA on chromosomes 3, 5, 8, 9 and Y, and the 45S rDNA was mapped in the pericentromeric region of the chromosome 4 short arm^15^. BAC-FISH mapped the BAC clones associated with the 12 linkage groups (LG) on the *C. papaya* pachytene chromosomes: BAC 96C17 (LG 1), BAC 39C20 (LG 9) and BAC 39P03 (LG 11), BAC 23B18 (LG 6 ), BAC 15O14 (LG 2), BAC 57E17 (LG 5), BAC 07H21 (LG 3), BAC 12M21 (LG 7) and BAC 01P02 (LG 12), BAC 43N18 (LG 4), BAC 78D03 (LG 8) and BAC 99D21 (LG 10), respectively on chromosomes 1 (XY), 2, 3, 4, 5, 6, 7, 8 and 9, integrating the LG to the individual chromosomes^15^.

In plants, physical mapping has contributed to the characterization of the sex chromosomes. The mapping of a repetitive DNA sequence, named HSR1, in *Humulus lupulus* L. showed that this sequence is located in the subtelomeric region of the X and Y chromosome long arm. However, the HSR1 was also mapped in the pericentromeric portion of the chromosome X^18^. In *Cannabis sativa* L., the repetitive sequence named CS-1 was mapped in the subtelomeric region of the Y chromosome short arm, while both arms of the X chromosome showed this sequence in the subtelomeric region^19^. The mapping of repetitive sequences in *Hippophae rhamnoides* L. revealed the HRTR 12 repetitive sequence only in the Y chromosome^20^. Repetitive DNA sequences, named RAYS, were only mapped in the Y chromosome of *Rumex acetosa* L.^21^. Therefore, the sex chromosome characterization in plants has been conducted mainly from the repeatome sequences. In addition to physical mapping, FISH has been accomplished to assists in the early identification of sporophyte sex. For example, a polymorphic molecular marker named NAPF-2 showed fluorescent signals in leaf nuclei of hermaphrodite plants, however, no signal was detected in leaf nuclei of female plants of *C. papaya* ‘Golden’ and ‘Rubi’^22^.

In addition to these sequences (mainly from the repeatoma), the physical mapping of single-copy and/or low-copy genes is needed to expand the knowledge about the *C. papaya* genome, contributing to knowing and understanding its organization and evolution. Moreover, these genes also have potential as cytomolecular markers. In this context, some sequences that occur in the sexual differentiation region were explored here, including: *somatic embryogenesis receptor kinase gene* (*serk 2*), *short vegetative phase gene* (*svp-like*) and *monodehydroascorbate reductase gene* (*mdar 4*)^12,23^.

The *serk 2* gene, which was sequenced in the X and Yh chromosomes^14^, encodes a protein that belongs to the plasma membrane receptor kinase family. SERK protein has leucine-rich repeats, acting on signal transduction^23^ and male sporogenesis^24^. The *svp-like* gene encodes a transcription factor that regulates the transition from the vegetative to the reproductive phase, activating the classes B and C genes of the floral morphogenesis^25,26^. Gene expression and sequencing approaches showed that the *svp-like* gene present on the Y chromosome encodes a wild-type protein with all domains. Due to a Copia-like retrotransposon insertion, a mutant allele of this gene occurs on the Yh chromosome, encoding a protein that has only the K-box domain^12,13,27,28^. In addition, *svp-like* gene was also identified on the chromosome 4^14^. The *mdar 4* gene, which was also sequenced in X and Yh chromosomes^14^, encodes an enzyme that has antioxidant activity, eliminating reactive oxygen species and, consequently, increasing tolerance against oxidative stress in some plant species^29,30^. Genetic analyzes have shown that the *mdar 4* gene has a wild-type allele on the X chromosome, while the allele of the Y and Yh chromosomes contains a LTR-retroelement sequence^30^. Y and Yh mutant alleles encode a shorter truncated MDAR 4 enzyme in flower tissues^28^.

Considering the previous genomic and cytogenomic data, we hypothesize that *C. papaya* sex chromosomes differ in several DNA sequences^31^, including *svp-like* and *mdar 4* genes. Based on this, we aimed to map the *serk 2*, *svp-like* and *mdar 4* sequences in *C. papaya* mitotic chromosomes. So, we start the mapping of single-copy and/or low-copy genes, differing from the previous *C. papaya* cytogenomics that were accomplished from rDNA genes and BAC sequences.

## Results

**Figure 1:**
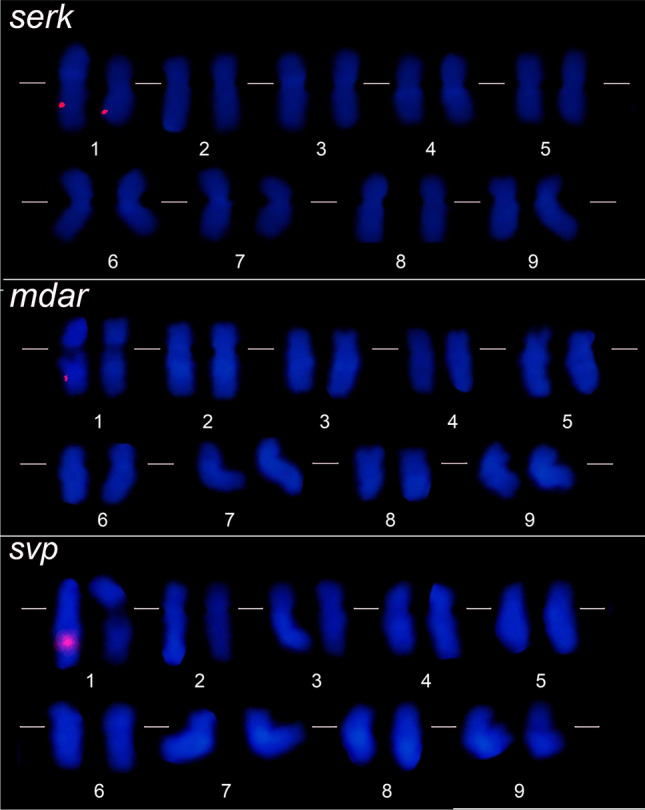
Physical mapping of the *serk 2*, *mdar 4* and *svp-like* sequences in mitotic chromosomes of *C. papaya*. Detail for the markings of the *serk 2*, *mdar 4* and *svp-like* in the interstitial region of the chromosome 1 long arm. The karyograms show 2n = 2x = 18 chromosomes, aligned by the centromere. The secondary constriction present in the pericentromeric region of chromosome 1 is observed. Bars: 10 μm.

The immersion of seeds in GA_3_ solution provided a high germination rate (95%) after ~ 5 days at 30 °C. Break seed dormancy was essential to increase the germination rate in a short period, providing a large amount of root meristems to obtain mitotic cells. Prometaphases and metaphases with morphologically preserved chromosomes exhibiting well-defined primary and secondary constrictions were obtained from the cytogenetics procedure. Therefore, we considered adequate the root meristem treatment with amiprophos-methyl/dimethyl sulfoxide, the enzymatic maceration, and the slide preparation accomplishing macerated root dissociation and air drying. The prometaphases and metaphases showed 2n = 2x = 18 chromosomes, confirming the *C. papaya* diploidy. The mean total chromosome length ranged from 4.32 ± 1.53 µm (chromosome 1) to 2.70 ± 0.42 µm (chromosome 9). *Carica papaya* karyogram possesses four metacentric (1, 5, 7 and 8) and five submetacentric chromosomes (2, 3, 4, 6 and 9, SI Table 1). Secondary constriction was identified in the pericentromeric region of the chromosome 1 (Fig. 1).

Sequences with ~ 1,100, ~ 250 and ~ 700 bp were amplified from *C. papaya* genomic DNA and *serk 2*, *mdar 4* and *svp-like* primers, respectively (SI Fig. 1). The similarity of the amplified *serk* 2 sequence in relation to *C. papaya* ‘Sunset’ and ‘SunUp’ genomes^14^ was 85% for *sense* strand and 82% for *antisense* strand in relation to sequenced chromosome 1, and 83% for *sense* strand and 81% for *antisense* strand in relation to chromosome Yh. For *mdar 4* sequence, the similarity was 95% for *sense* strand and 94% for *antisense* strand in relation to the chromosome 1, and 90% for *sense* strand and 89% for *antisense* strand in relation to the chromosome Yh. The similarity of the *svp-like* sequence was 100% for *sense* strand and 99% for *antisense* strand in relation to the chromosome 4, and 86% for *sense* strand and 82% for *antisense* strand in relation to the chromosome Yh.

We defined the FISH procedure based on different tests and their respective results, considering the visualization of fluorescence signals in nuclei and mitotic chromosomes. We used 4X SSC in the post-hybridization wash solution (stringency). Thus, the FISH conditions were established for the *serk 2*, *svp-like* and *mdar 4* probes, which showed different sizes in bp. The hybridizations were performed in prometaphases and metaphases to map these sequences, and also in interphase nuclei to confirm the copy number of each sequence. So, clear signals were generated after hybridization of the probes in nuclei and chromosomes.

**Figure 2:**
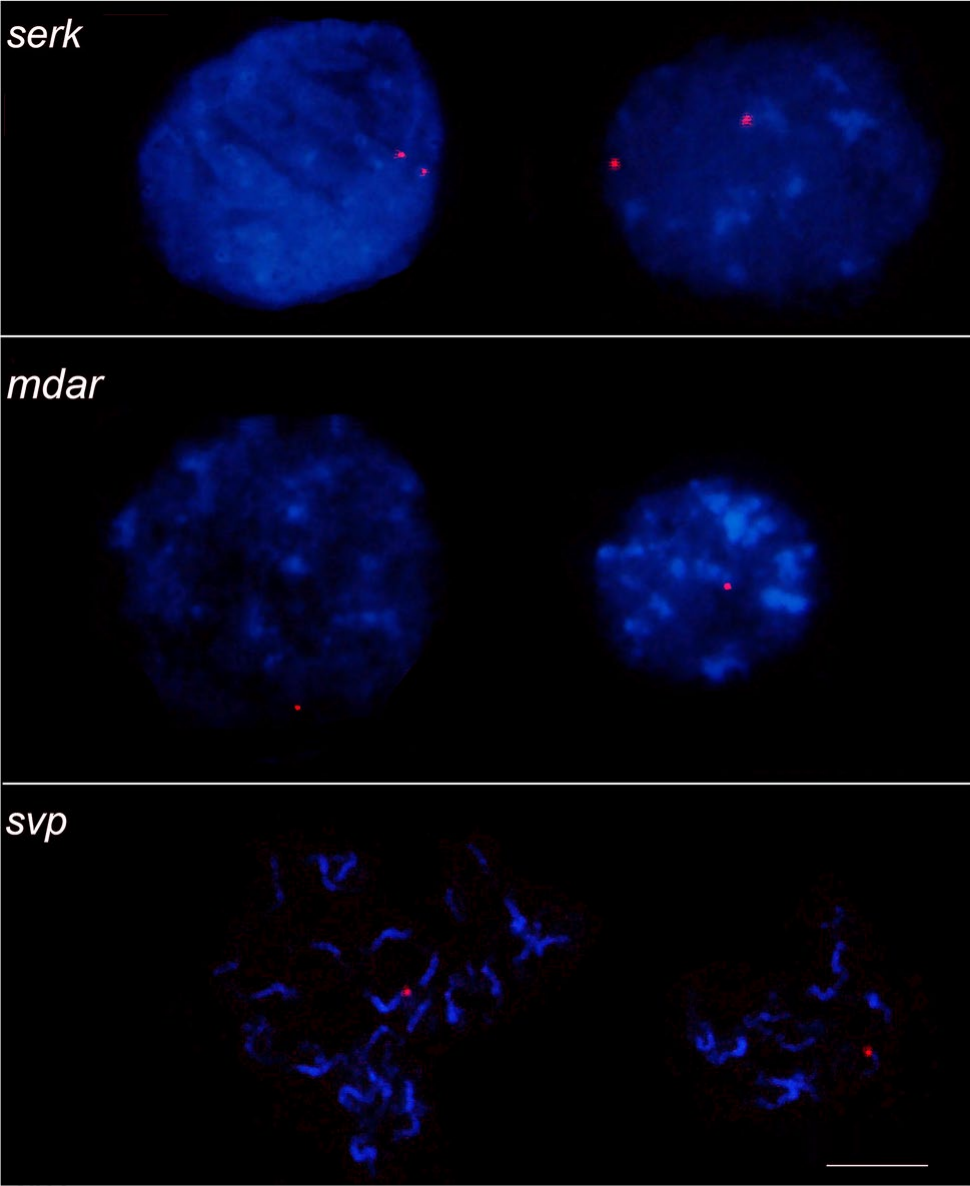
Copy number of the *serk 2*, *mdar 4* and *svp-like* sequences in interphase nuclei of *C. papaya*. Two signals were detected for *serk 2*, and one for *mdar 4* and *svp-like*.

The *serk 2* sequence was mapped in prometaphases/metaphases on the interstitial region of the long arm of chromosome 1 pair, which was identified from the secondary constriction in pericentromeric region. This chromosome has been appointed as the sex chromosome of *C. papaya*. In addition, two fluorescence signals were detected in interphase nuclei, confirming that there is one copy of the *serk 2* in the *C. papaya* genome. As well as *serk 2*, *svp-like* and *mdar 4* sequences were mapped in prometaphases/metaphases in the interstitial region of the long arm of chromosome 1. However, only one chromosome 1 exhibited these sequences (Figs. 1 and 2). Corroborating this, we found one *svp-like* and *mdar 4* fluorescence in interphase nuclei. Thus, our results suggest differences between the chromosomes of the homologous pair 1, also designated as X and Y/Yh.

**Figure 3:**
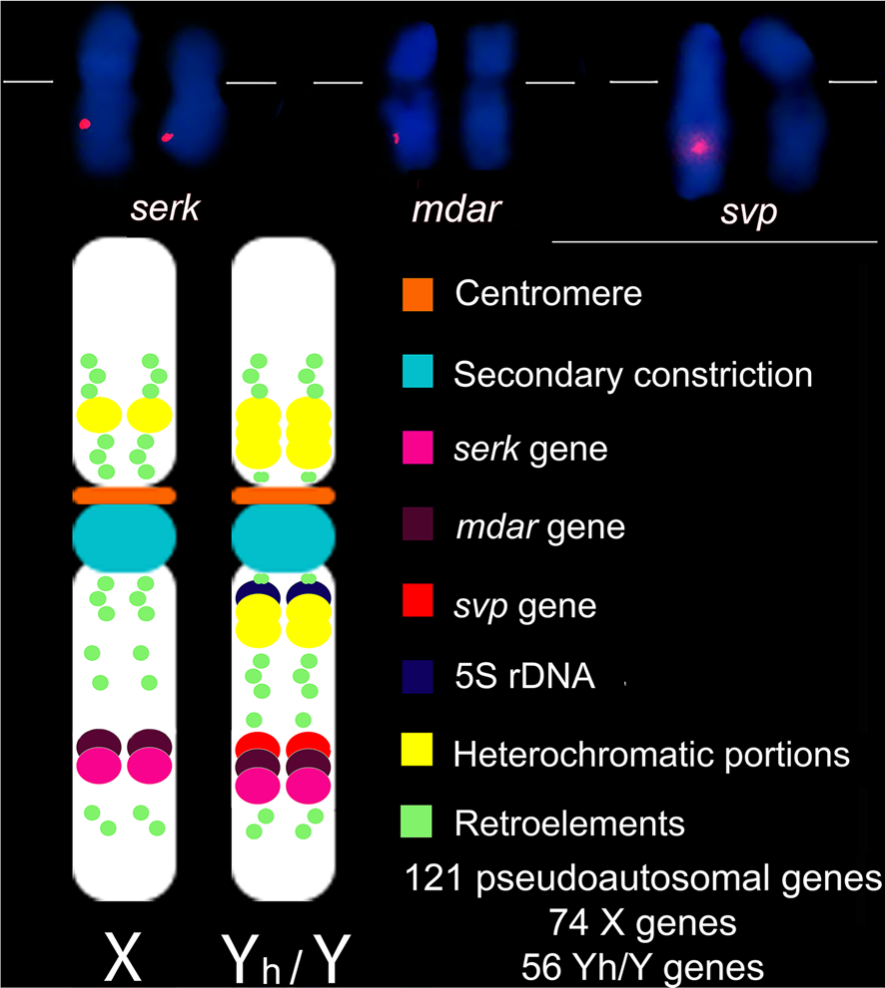
Ideograms of the X and Yh/Y chromosomes of *C. papaya*. Chromosomes 1 exhibiting the fluorescence signal for *serk 2*, *mdar 4* and *svp-like* sequences. The orange bar represents the position of the centromere. The light blue circle shows the position of the secondary constriction. The pink and purple circles on the long arm of the X chromosome show the loci of the *serk 2* and *mdar 4* sequences, respectively. The pink, purple and red circles on the long arm of the Yh/Y chromosome shows the loci of the *serk 2*, *mdar 4* and *svp-like* genes. The yellow circles represent the knobs (heterochromatic portions)^39^. The dark blue circle in the pericentromeric region of the long arm of the Y chromosome represents the 5S rDNA^15^. The green circles show the distribution of retroelements along the sex chromosomes^27^.

Based on the karyograms (Fig. 1), we structured an ideogram of the chromosomes X and Y/Yh, representing the loci marked by the *serk 2*, *mdar 4* and *svp-like*, as well as the centromere and the secondary constriction (Fig. 3). The results obtained in this study increase the cytogenomic data of *C. papaya* through the physical mapping of the *serk 2*, *mdar 4* and *svp-like*. In addition, the ideogram includes the cytogenomic data of the physical location of heterochromatic portions^32^, the 5S rDNA gene^15^ and the distribution of retroelements^27^.

## Discussion

We mapped the *serk 2*, *mdar 4* and *svp-like*, providing the first physical mapping of these sequences in *C. papaya* mitotic chromosomes. Accomplishing the same FISH procedure, we mapped amplicons from 251 to 1166 bp, resulting in a physical mapping in mitotic chromosomes from sequences of single-copy or low-copy in *C. papaya*. Our procedure can be reproduced allowing the physical mapping of genes with single-copy or low-copy. In addition, our results represent the start point for further physical mapping, which are relevant to increase knowledge about the *C. papaya* genome. We integrated the physical map with the in silico data, contributing to knowing and understanding of the sexual chromosomes X and Y/Yh of the *C. papaya*. In addition, the *svp-like* can be further used as cytomolecular markers to previously screen the sex of *C. papaya* plants.

*C. papaya* is a model species for studies about sex chromosome evolution and sex differentiation in plants, mainly due to incipient evolution of its sex chromosomes X and Y/Yh, and the recombination suppression in the non-homologue portion between them^1,5^. In addition to the *svp-like* and *mdar 4* sequences, a relatively high density of retroelements was identified along the *C. papaya* sex chromosomes^27^. Furthermore, at least four knobs (heterochromatic portions) and highly methylated sequences were identified only in the sexual region of the Y/Yh chromosome and not in the non-homologous portion of the X chromosome^32^. These cytogenomic characteristics may influence gene expression and sequence divergence.

*serk 2* physical mapping revealed its locus in the interstitial region of the chromosome 1 long arm. The sequencing of the X and Y/Yh sex chromosome dedifferentiation portions showed that the *serk 2* gene is present in this region^12,14^. The presence of two signals in the nuclei and in the metaphases confirms that the *serk 2* is a single-copy gene, and that it possibly occurs near or in the pseudoautosomal portion of the X and Y/Yh chromosomes. Although the sex determination region has specific genes or sequences of each sex chromosome (74 genes in X, and 56 genes in Yh), at least 121 genes have been predicted in the sex region of the X chromosome and in its Yh counterpart^13,14^.

*mdar 4* sequence, which was amplified from *C. papaya* genomic DNA, locus was also mapped in the chromosome 1 interstitial region, and only one hybridization signal of this sequence was detected in the nuclei and chromosomes. Genomic analyses showed that the divergence between the *mdar 4* gene in sex chromosomes is due to the insertion of retroelements inside of this gene in Y (male) and Yh (hermaphrodite) chromosomes, which does not occur in X chromosome^27^. Since the *mdar 4* gene plays a role in eliminating reactive oxygen species, maintaining cell viability, the absence of this gene on the X chromosome can be lethal^30^. The alignment of a region of the X and Yh *C. papaya* chromosomes revealed deletions on the Yh. For example, the *asymmetric leaves 2* gene appears to have been lost, since it occurs only on the X chromosome^10^. Gene loss has been reported in *Silene latifolia* Poir. Y chromosome, which lossed ~ 14.5 of genes^33^. Thus, further studies in *C. papaya*, including cytogenomics, need to be conducted to show possible gene loss between the X and Y/Yh chromosomes.

*svp-like* was mapped in the interstitial region of the 1 chromosome long arm. The transcriptome indicated that *svp-like* gene occurs only on the sex differentiation region of the Y (male) and Yh (hermaphrodite) chromosome^27^. *svp-like* gene was also identified by sequencing in the chromosome 4^14^. The sequencing showed that our *svp-like* sequence possesses similarity in relation to *svp-like* gene of the Yh chromosome. Therefore, our data suggest that the *svp-like* sequence was mapped in the Y/Yh chromosome.

The mapping of the *svp-like* and *mdar 4* sequences on chromosome 1, which shows the secondary constriction and the higher total length. So, this result corroborates with sex differentiation region in *C. papaya*, which is in a pericentromeric region that expands to the long arm of this chromosome^33^. The expansion of the suppressed recombination region occurs from the selection of sexual antagonistic genes and/or chromosomal rearrangements^33–35^. As a consequence of the recombination suppression in the sex differentiation region, the Y/Yh chromosome undergoes genetic degeneration, presenting low expression or loss of genes. Suppressed recombination in sex differentiation region can promote divergence between the sequences present in this chromosomal portion and gradually change its copy number.

The identification of cytogenetic differences and the characterization of sex chromosomes in plants have been conducted mainly through physical mapping of repetitive sequences. In *H. lupulus*, the satellite DNA HSR1 was mapped in the subtelomeric and pericentromeric region of the X chromosome, while the Y chromosome presented this sequence only in the subtelomeric region^18^. The mapping of the repetitive sequence CS-1 differentiated the sex chromosomes of *C. sativa*, due to this sequence occurs only in the subtelomeric region of the Y chromosome short arm and in the subtelomeric region of both arms of the X chromosome^18^. In *H. rhamnoides* and *R. acetosa*, the satellite DNA sequences HRTR 12 and RAYS, respectively, were mapped only on the Y chromosome^20,21^.

In addition to the sex chromosomes characterization, molecular cytogenetics have been accomplished to determine the plant sex before the reproductive period. Based on our results and previous sequencing data, the *svp-like* sequence can be explored as cytomolecular markers to early determine the sex of *C. papaya* plants. In *C. papay*a, molecular markers of the *Sequence Characterized Amplified Region* type (SCAR) were used to differentiate hermaphrodite individuals from females, through the FISH application in nuclei isolated from the leaves. Using the molecular marker NAPF-2, only the nuclei isolated from the hermaphrodite plants showed a strong fluorescence signal, which was not observed in the leaf nuclei of female plants^22^. Thus, FISH is also useful to early determine and screen the sex, reducing the growing costs of the sex of noncommercial interest.

For the first time, single-copy sequences were mapped in *C. papaya* sex chromosomes. We mapped *serk 2*, *svp-like* and *mdar 4* sequences, which are single-copy or low-copy sequences, in mitotic chromosomes of *C. papaya*. Therefore, our data represents the start point to map other specific sex chromosome genes, increasing cytogenomics analyzes and the understanding of the evolution and structure of the sex chromosomes in *C. papaya*.

## Material and methods

Commercial *C. papaya* ‘Hawaii’ (Isla^®^), a gynodioecious line, seeds were bought in accordance to relevant guidelines and legislation. We declare that all methods were performed in accordance with the relevant guidelines.

### Data availability

The datasets analysed for *serk 2*, *svp-like* and *mdar 4* sequences during the current study are available in: https://www.ncbi.nlm.nih.gov/gene/?term=LOC110813265 for *serk 2*, https://www.ncbi.nlm.nih.gov/nuccore/XM_022047757.1 for *svp-like*, and https://www.ncbi.nlm.nih.gov/gene/?term=LOC110807062 for *mdar 4*, as well as https://www.nature.com/articles/s41588-022-01068-1, and https://ngdc.cncb.ac.cn/gwh/ GWHBFSC00000000 for *C. papaya* ‘SunUp’ and GWHBFSD00000000 for *C. papaya* ‘Sunset’^14^ for the three genes. The chromosome morphometry data generated and analysed during this study are included in this published article and its supplementary information files.

### Mitotic chromosomes

Commercial seeds of *C. papaya* ‘Hawaii’ (Isla^®^) were immersed in 200 ppm gibberellic acid (GA_3_) for 24 h to break seed dormancy^36^. Subsequently, the seeds were placed in Petri dishes with filter paper moistened with dH_2_O and kept at 30 °C until germination. The 0.5–1.0 cm long roots were treated with 3 µM amiprophos-methyl (Sigma^®^) and 0.3% dimethyl sulfoxide (Sigma^®^) for 4 h at 30 °C. The roots were washed in dH_2_O and fixed in methanol:acetic acid (3:1), with three changes of 10 min each, and kept at − 20 °C^37,38^. Root meristems were excised, washed in dH_2_O three times, and incubated at 36 °C for 2 h in an enzyme solution (4% cellulase Sigma^®^, 0.4% hemicellulase Sigma^®^, 1% macerozyme R10 Yakult^®^, 100% pectinase Sigma^®^) diluted in dH_2_O at 1:14 (enzymatic solution: dH_2_O). After enzymatic maceration, root meristems were washed in dH_2_O, fixed in methanol:acetic acid (3:1) and kept at − 20 °C. The slides were prepared using cell dissociation and air-drying techniques^3^.

### FISH

*Carica papaya* total genomic DNA was extracted from young leaves using GenElute™ Plant Genomic DNA Miniprep Kit (Sigma^®^), following the manufacturer's instructions. DNA concentration and purity were determined by a NanoDrop 1000 spectrophotometer (Thermo Fisher Scientific^®^), and its integrity was evaluated by electrophoresis in a 1.5% agarose gel. The primers of the *serk 2* and *mdar 4* were designed based on the sequences deposited at the National Center for Biotechnology Information (NCBI): *serk 2*—LOC110813265 and *mdar 4*—LOC110807062. For the *svp-like*, the primers were obtained based on the bibliography^34^. So, we designed the primers: *F* 5′-CTCTCACTGCACGCCTAAC-3′ and *R* 5′-TCGCCTTCAAATCCTGAAACT-3' for *serk 2* providing a 1,166 bp amplification product; *F* 5′-ACTTGTTGCCTCAGTTTCTCATTCTCTTC-3′ and *R* 5′-GAGATCAGTGATCTTCAAAGGAAGGTC-3′ for *svp-like* resulting a 251 bp amplification product; and *F* 5′-TATTCCGACCCCAGTCTCCA-3' and *R* 5'-TCCTACCGCGCCAAACAAAT-3′ for *mdar 4* providing a 731 bp amplification product. The primers were validated in silico using the OligoAnalyzer™ tool (https://www.idtdna.com/pages/tools/oligoanalyzer), and the formation of homodimers, heterodimers and hairpin structures were verified. Specificity analysis was performed using the Basic Local Alignment Search Tool (BLAST, https://blast.ncbi.nlm.nih.gov/Blast.cgi).

PCR reactions for amplification the gene sequences consisted of 0.5 μM of each primer, 60 ng genomic DNA, 200 μM of each dNTP (Promega^®^), 1.3 mM MgCl_2_ (Promega^®^), 1X Colorless GoTaq^®^ Flexi Buffer (Promega^®^) and 1 U of the enzyme GoTaq^®^ Flexi DNA Polymerase (Promega^®^). All amplifications were performed in a PTC-200 Peltier Thermal Cycler (MJ Research, Inc) under the following conditions: 4 min at 95 °C, followed by 30 cycles of 1 min at 95 °C, 1 min at 54 °C, 50 °C and 50 °C, respectively, for the *serk 2*, *svp-like* and *mdar 4* primers, 1 min at 72 °C, and 5 min at 72 °C for a final extension. PCR products were analyzed on 1.5% agarose gel (SI Fig. 1).

PCR reactions for probe labeling were accomplished from amplified gene fragments. The reactions consisted of 0.5 μM of each primer, 200 ng of previously amplified sequences, 200 μM of each dATP, dCTP and dGTP (Promega^®^), 100 μM of dTTP (Promega^®^), 20 μM Tetramethylrhodamine-5-dUTP (Roche^®^), 1.3 mM MgCl_2_, 1X Colorless GoTaq^®^ Flexi Buffer (Promega^®^) and 1 U of the enzyme GoTaq^®^ Flexi DNA Polymerase (Promega^®^)^38^. The PCR conditions were the same used for the gene sequences amplification.

Slides with prometaphases and metaphases exhibiting chromosomes with well-defined centromeres were selected from phase contrast microscope Olympus BX41. The selected slides were treated in 1X phosphate-buffered saline (PBS) for 5 min, fixed in 4% formalin solution for 10 min, and washed with 1X PBS for 5 min. Subsequently, the slides were dehydrated in an ice-cold alcoholic series (70%, 85% and 100%) for 3 min each. The hybridization mix consisted of 200 ng of each probe, 50% formamide and 2X saline sodium citrate (SSC). Chromosomes were denatured in a water bath at 70 °C for 3 min in 70% formamide/2X SSC. After denaturation, the slides were dehydrated in an ice-cold alcoholic series (70%, 85% and 100%) for 3 min each. The hybridization mix was denatured in a PTC-200 Peltier Thermal Cycler (MJ Research, Inc) at 85 °C for 5 min and immediately placed on ice for at least 5 min. The hybridization mix was placed on the slides, covered with HybriSlip™ plastic coverslips (Sigma^®^) and sealed with Rubber Cement glue (Elmer’s^®^). Hybridization was carried out in the ThermoBrite™ equipment (StatSpin^®^) at 37 °C for 24 h. Subsequently, the slides were submitted to a stringency wash performed at 40 °C in 4X SSC solution for 5 min. Slides were counterstained with 10% glycerol/PBS + 4′,6-diamidino-2-phenylindole (DAPI), covered with a glass coverslip and sealed with nail polish^39–42^. Nuclei, prometaphases and metaphases were captured with a 12-bit CCD digital camera (Olympus^®^) attached to an Olympus BX-60 photomicroscope equipped with epifluorescence and 100 × PlanApo immersion objective (NA = 1.4). Captured images were processed using Image-Pro Plus 6.1 software (Media Cybernetics, Inc).

### Sequencing of the amplification products from *serk 2*, *svp-like* and *mdar 4* primers

PCR reactions were performed with a total volume of 50 μL and consisted of 0.5 μM of each primer, 200 ng of previously amplified sequences, 200 μM of each dNTP (Promega^®^), 1.3 mM of MgCl_2_, 1X of Colorless GoTaq^®^ Flexi Buffer (Promega^®^) and 1 U of the enzyme GoTaq^®^ Flexi DNA Polymerase (Promega^®^). PCR conditions were the same used for amplification of the sequences from *serk 2*, *svp-like* and *mdar 4* primers. The purification of the amplified sequences was performed with the Wizard^®^ SV Gel and PCR Clean-Up System (Promega^®^) kit, following the manufacturer’s instructions. Concentration and purity of the PCR products were determined by a NanoDrop 1000 spectrophotometer (Thermo Fisher Scientific^®^), and their integrity was evaluated by electrophoresis in a 1.5% agarose gel. Purified PCR products were sent for sequencing by the Sanger method at ACTGene Análises Moleculares.

From sequenced *serk 2*, *svp-like* and *mdar 4*, we verified the similarity of these sequences in relation to the *C. papaya* ‘SunUp’ (JAIUCH000000000, https://www.ncbi.nlm.nih.gov/nuccore/JAIUCH010000010.1) and *C. papaya* ‘Sunset’ (JAIUCG000000000, https://www.ncbi.nlm.nih.gov/nuccore/JAIUCG010000010.1) genomes^14^. We evaluated the similarity of our amplified *serk 2*, *svp-like* and *mdar 4* sequences in relation to each *C. papaya* chromosome: chromosome 1 that contains the X and Yh sexual differentiation regions, chromosomes 2–9, and chromosome Yh (SI Genomic Data).

### Supplementary Information

Supplementary Figure 1.

Supplementary Information.

Supplementary Table 1.

## Data Availability

All data generated and analysed during this study are included in this published article and its supplementary information files.
