# Supplementary material for: Carica papaya L. sex chromosome review and physical mapping of the serk 2, svp-like and mdar 4 sequences
Source: Sci Rep. 2024 Jun 27;14:14830. doi: 10.1038/s41598-024-65880-x (PMC11211501; doi:10.1038/s41598-024-65880-x)
Supplement: Supplementary file 1 — Supplementary Figure 1. [file 41598_2024_65880_MOESM1_ESM.pdf]

Title: *Carica papaya* L. sex chromosome review and physical mapping of the *serk 2*, *svp-like* and *mdar 4* sequences

Authors: Adeilson Frias Dornela[1], Fernanda Aparecida Ferrari Soares[2], Jéssica Coutinho Silva[2], Mariana Cansian Sattler[2], Wellington Ronildo Clarindo[2].

[1]Pós-graduação em Genética e Melhoramento, Centro de Ciências Agrárias e Engenharias, Universidade Federal do Espírito Santo. ZIP: 29.500-000, Alegre – ES, Brazil.

[2]Laboratório de Citogenética e Citometria, Departamento de Biologia Geral, Centro de Ciências Biológicas e da Saúde, Universidade Federal de Viçosa. ZIP: 36.570-900, Viçosa – MG, Brazil.

Corresponding author: e-mail: well.clarindo@ufv.br

Tel.: +55 31 3612-5028

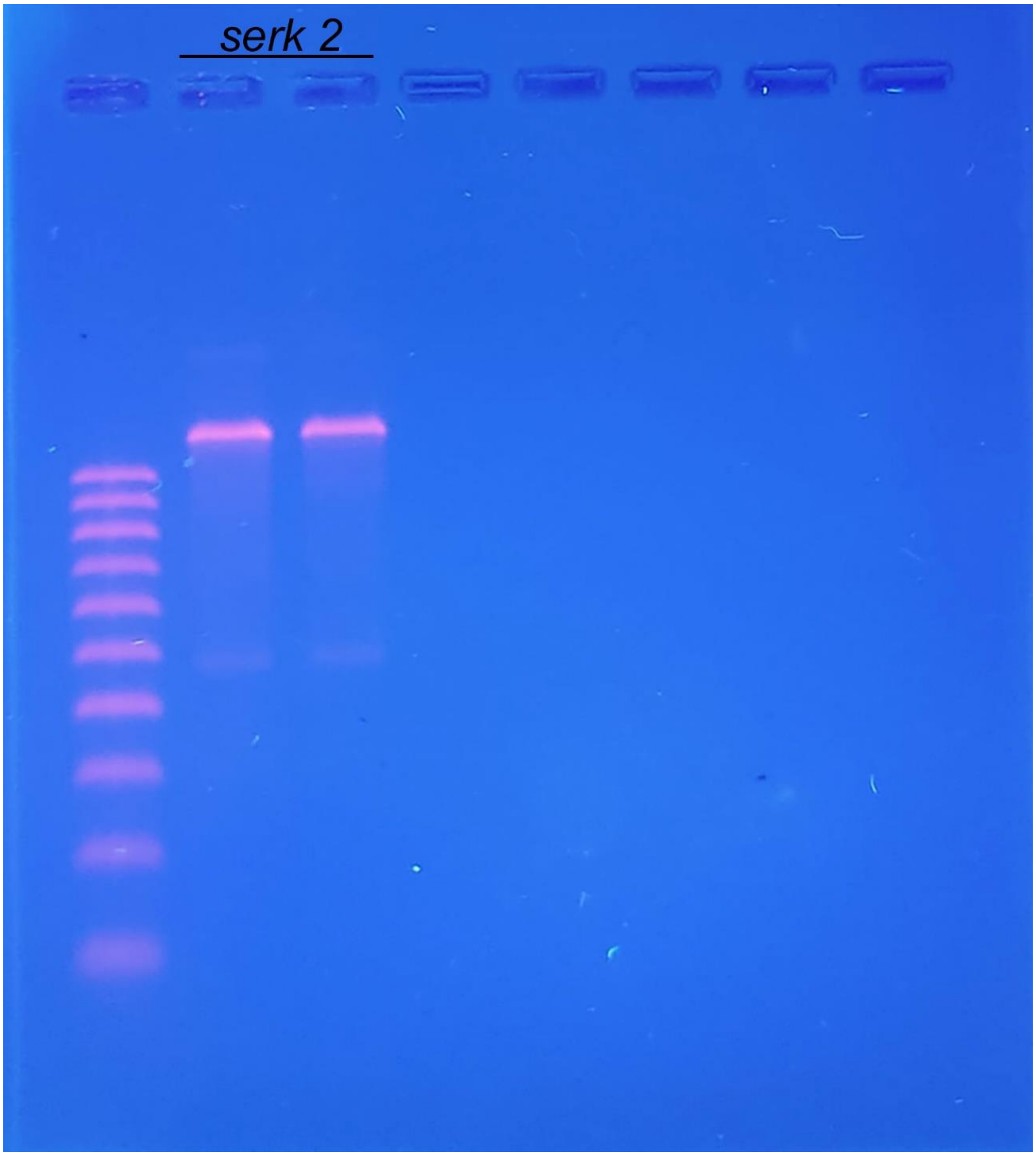

SI Figure 1a. Amplified products, which were analyzed in 1.5% agarose gel, from PCR of *C. papaya* genomic DNA using specific primers for *serk 2* gene. *serk 2* primers (*F* 5'-CTCTCACTGCACGCCTAAC-3' and *R* 5'-TCGCCTTCAAATCCTGAAACT-3') provided a 1,166 bp amplification product, which was used to construct the probe.

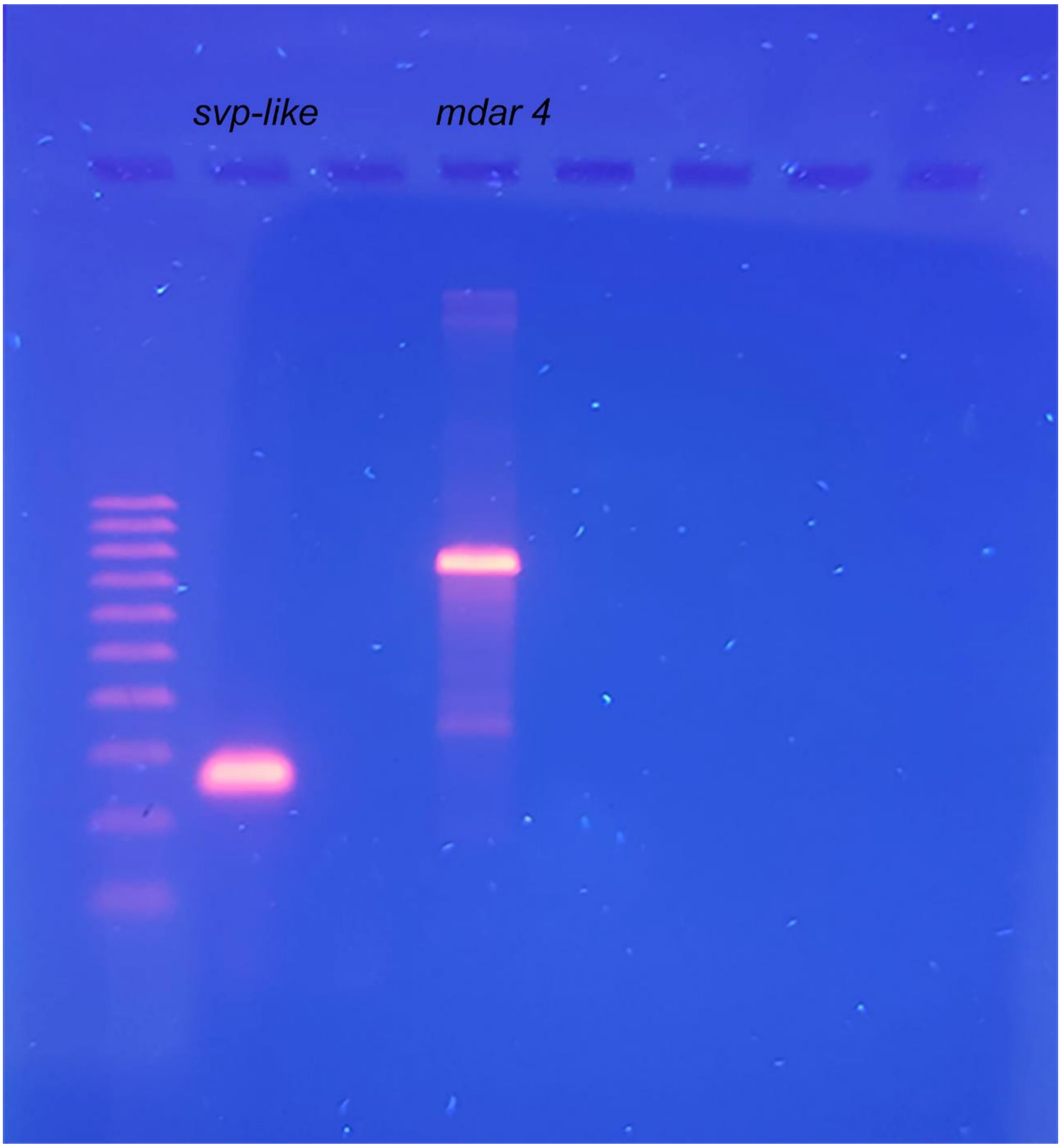

SI Figure 1b. Amplified products, which were analyzed in 1.5% agarose gel, from PCR of *C. papaya* genomic DNA using specific primers for *svp-like* and *mdar 4*, respectively. *svp-like* (*F* 5'-ACTTGTTGCCTCAGTTTCTCATTCTCTTC-3' and *R* 5'-GAGATCAGTGATCTTCAAAGGAAGGTC-3') and *mdar 4* (*F* 5'-TATTCCGACCCCAGTCTCCA-3' and *R* 5'-TCCTACCGCGCCAAACAAAT-3') primers resulted, respectively, in 251 bp and 731 bp amplification products, which were used to construct the probes.
