# Supplementary material for: Carica papaya L. sex chromosome review and physical mapping of the serk 2, svp-like and mdar 4 sequences
Source: Sci Rep. 2024 Jun 27;14:14830. doi: 10.1038/s41598-024-65880-x (PMC11211501; doi:10.1038/s41598-024-65880-x)
Supplement: Supplementary file 2 — Supplementary Information. [file 41598_2024_65880_MOESM2_ESM.pdf]

Supplementary genomic data

We compared our amplified *serk 2*, *svp-like* and *mdar 4* sequences in relation to the with deposited data[14].

1. We used the two genomes available[14]. The genome accession number of the 'SunUp' is **JAIUCH000000000** and the 'Sunset'is **JAIUCG000000000**.

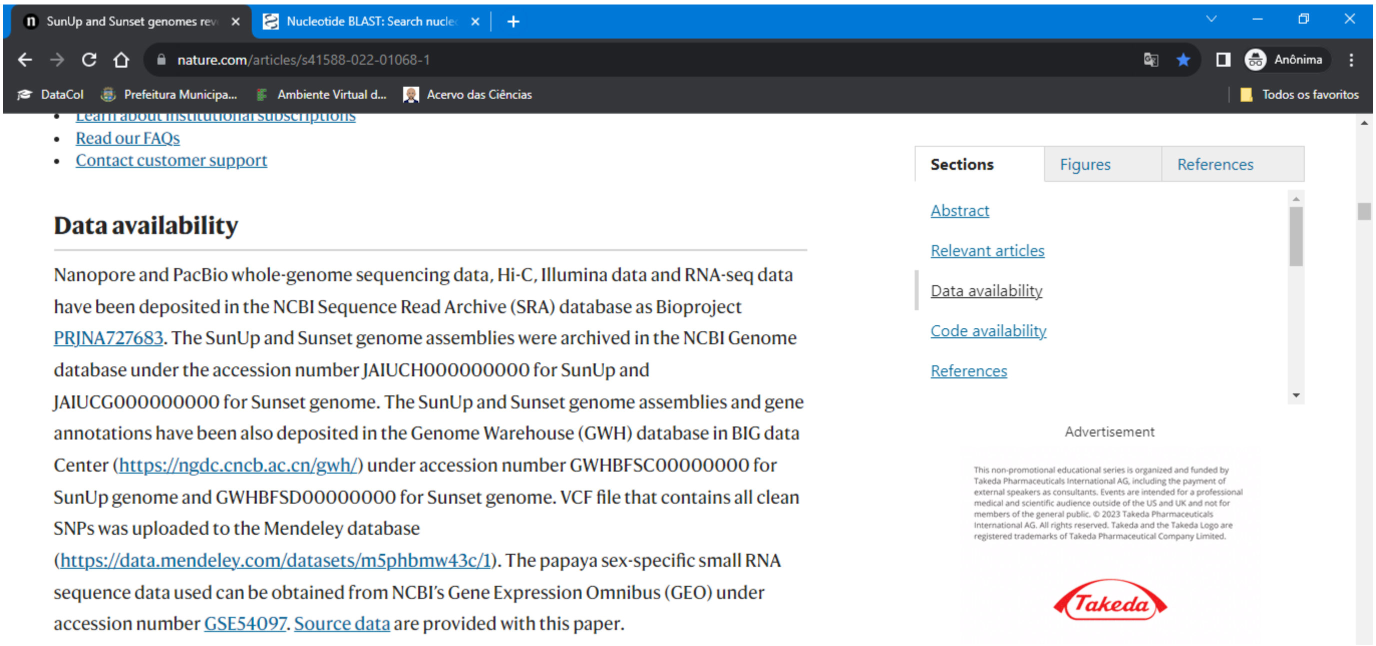

2. For example, we used the 8043405 bp genomic data available from ‘SunUp’ – https://www.ncbi.nlm.nih.gov/nuccore/JAIUCH010000010.1

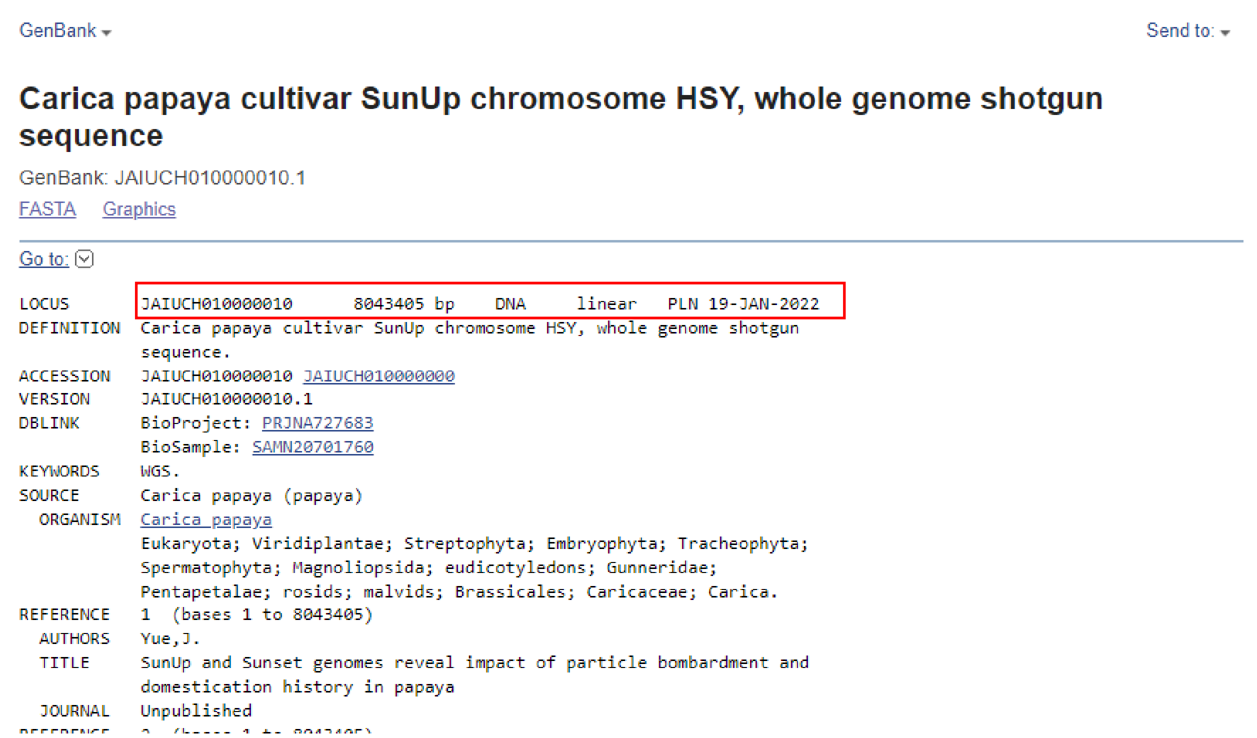


```
LOCUS       JAIUCH010000010      8043405 bp    DNA     linear   PLN 19-JAN-2022
DEFINITION  Carica papaya cultivar SunUp chromosome HSY, whole genome shotgun
            sequence.
ACCESSION   JAIUCH010000010 JAIUCH010000000
VERSION     JAIUCH010000010.1
DBLINK      BioProject: PRJNA727683
            BioSample: SAMN20701760
KEYWORDS    WGS.
SOURCE      Carica papaya (papaya)
  ORGANISM  Carica papaya
            Eukaryota; Viridiplantae; Streptophyta; Embryophyta; Tracheophyta;
            Spermatophyta; Magnoliopsida; eudicotyledons; Gunneridae;
            Pentapetalae; rosids; malvids; Brassicales; Caricaceae; Carica.
REFERENCE   1  (bases 1 to 8043405)
  AUTHORS   Yue,J.
  TITLE     SunUp and Sunset genomes reveal impact of particle bombardment and
            domestication history in papaya
  JOURNAL   Unpublished
```

3. After, we attached the sequenced amplification to verify the similatiry.

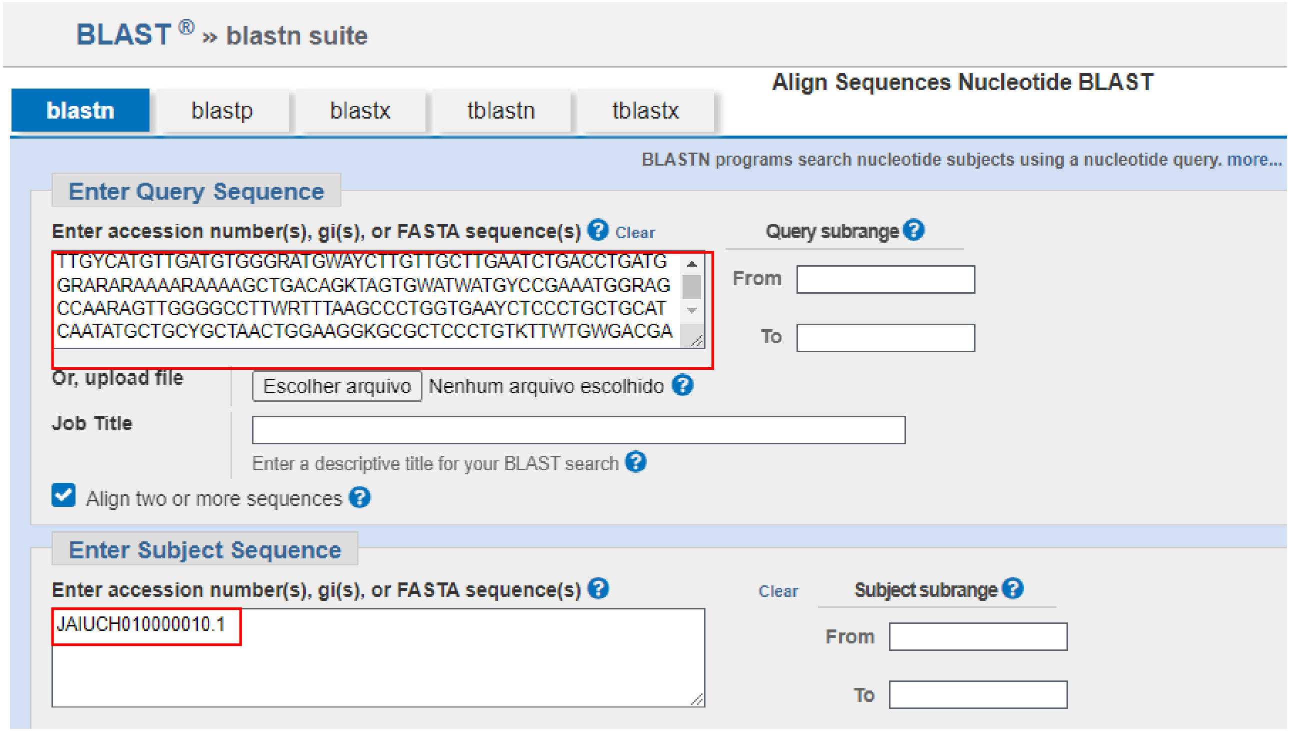

4. Genomic similarity – BLAST results

	C. papaya 'Sunset'						*C. papaya* 'SunUp'					
	serk 2		*mdar 4*		*svp-like*		*serk 2*		*mdar 4*		*svp-like*	
	F	*R*	*F*	*R*	*F*	*R*	*F*	*R*	*F*	*R*	*F*	*R*
Chromosome 1	**85%**	**82%**	**95%**	**94%**	0%	0%	**85%**	**82%**	**95%**	**94%**	0%	0%
Chromosome 2	0%	0%	0%	0%	0%	0%	0%	0%	0%	0%	0%	0%
Chromosome 3	0%	0%	0%	0%	0%	0%	0%	0%	0%	0%	0%	0%
Chromosome 4	0%	0%	0%	0%	**100%**	**99%**	0%	0%	0%	0%	**100%**	**99%**
Chromosome 5	0%	0%	0%	0%	0%	0%	0%	0%	0%	0%	0%	0%
Chromosome 6	0%	0%	0%	0%	0%	0%	0%	0%	0%	0%	0%	0%
Chromosome 7	0%	0%	0%	0%	0%	0%	0%	0%	0%	0%	0%	0%
Chromosome 8	0%	0%	0%	0%	0%	0%	0%	0%	0%	0%	0%	0%
Chromosome 9	0%	0%	0%	0%	0%	0%	0%	0%	0%	0%	0%	0%
Chromosome HSY	**83%**	**81%**	**90%**	**89%**	**86%**	**82%**	**83%**	**80%**	**90%**	**89%**	**86%**	**82%**
