# Supplementary material for: Carica papaya L. sex chromosome review and physical mapping of the serk 2, svp-like and mdar 4 sequences
Source: Sci Rep. 2024 Jun 27;14:14830. doi: 10.1038/s41598-024-65880-x (PMC11211501; doi:10.1038/s41598-024-65880-x)
Supplement: Supplementary file 3 — Supplementary Table 1. [file 41598_2024_65880_MOESM3_ESM.pdf]

SI Table 1. Morphometry and classification of *C. papaya* chromosomes.

Chromosome	Total length (µm)	Arms (µm)		r	i	Relative length (%)	Class
		Short	Long				
1	4.32 ± 1.53	1.79 ± 0.69	2.53 ± 1.16	1.42	41.4	5.56	M
2	3.72 ± 0.90	1.43 ± 0.52	2.29 ± 0.41	1.60	38.5	4.79	SM
3	3.54 ± 0.84	1.17 ± 0.20	2.37 ± 0.75	2.04	32.9	4.55	SM
4	3.38 ± 0.87	1.25 ± 0.39	2.13 ± 0.24	1.70	37.0	4.35	SM
5	3.27 ± 0.70	1.34 ± 0.27	1.93 ± 0.29	1.44	41.0	4.21	M
6	3.22 ± 0.70	1.20 ± 0.30	2.02 ± 0.38	1.68	37.3	4.14	SM
7	3.17 ± 0.64	1.29 ± 0.35	1.88 ± 0.31	1.46	40.6	4.08	M
8	2.81 ± 0.42	1.16 ± 0.42	1.66 ± 0.25	1.43	41.1	3.62	M
9	2.70 ± 0.42	1.08 ± 0.27	1.63 ± 0.15	1.51	39.9	3.48	SM
	30.14	11.70	18.44			38.78	

r: arm ratio, i: centromeric index, M: metacentric, SM: submetacentric
